# An Intraoperative Visualization System Using Hyperspectral Imaging to Aid in Brain Tumor Delineation

**DOI:** 10.3390/s18020430

**Published:** 2018-02-01

**Authors:** Himar Fabelo, Samuel Ortega, Raquel Lazcano, Daniel Madroñal, Gustavo M. Callicó, Eduardo Juárez, Rubén Salvador, Diederik Bulters, Harry Bulstrode, Adam Szolna, Juan F. Piñeiro, Coralia Sosa, Aruma J. O’Shanahan, Sara Bisshopp, María Hernández, Jesús Morera, Daniele Ravi, B. Ravi Kiran, Aurelio Vega, Abelardo Báez-Quevedo, Guang-Zhong Yang, Bogdan Stanciulescu, Roberto Sarmiento

**Affiliations:** 1 Institute for Applied Microelectronics (IUMA), University of Las Palmas de Gran Canaria (ULPGC), Las Palmas de Gran Canaria 35017, Spain; sortega@iuma.ulpgc.es (S.O.); gustavo@iuma.ulpgc.es (G.M.C.); avega@iuma.ulpgc.es (A.V.); abaez@iuma.ulpgc.es (A.B.-O.); roberto@iuma.ulpgc.es (R.S.); 2 Centre of Software Technologies and Multimedia Systems (CITSEM), Technical University of Madrid (UPM), Madrid 28031, Spain; raquel.lazcano@upm.es (R.L.); daniel.madronal@upm.es (D.M.); ejuarez@sec.upm.es (E.J.); ruben.salvador@upm.es (R.S.); 3 Wessex Neurological Centre, University Hospital Southampton, Tremona Road, Southampton SO16 6YD, UK; dbulters@nhs.net; 4 Department of Neurosurgery, Addenbrookes Hospital and University of Cambridge, Cambridge CB2 0QQ, UK; hb252@cam.ac.uk; 5 Department of Neurosurgery, University Hospital Doctor Negrin, Las Palmas de Gran Canaria 35010, Spain; adamszolna@wp.pl (A.S.); pinerbrains1@yahoo.es (J.F.P.); coralia.sosa@gmail.com (C.S.); aruosha@gmail.com (A.J.O.); sarabisshop@hotmail.com (S.B.); hhdez.maria@gmail.com (M.H.); jmormol@gobiernodecanarias.org (J.M.); 6 The Hamlyn Centre, Imperial College London (ICL), London SW7 2AZ, UK; d.ravi@imperial.ac.uk (D.R.); g.z.yang@imperial.ac.uk (G.-Z.Y.); 7 Laboratoire CRISTAL, Université Lille 3, Villeneuve-d’Ascq 59653, France; ravi.kiran@esiee.fr; 8 Ecole Nationale Supérieure des Mines de Paris (ENSMP), MINES ParisTech, Paris 75006, France; bogdan.stanciulescu@mines-paristech.fr

**Keywords:** hyperspectral imaging instrumentation, brain cancer detection, image processing

## Abstract

Hyperspectral imaging (HSI) allows for the acquisition of large numbers of spectral bands throughout the electromagnetic spectrum (within and beyond the visual range) with respect to the surface of scenes captured by sensors. Using this information and a set of complex classification algorithms, it is possible to determine which material or substance is located in each pixel. The work presented in this paper aims to exploit the characteristics of HSI to develop a demonstrator capable of delineating tumor tissue from brain tissue during neurosurgical operations. Improved delineation of tumor boundaries is expected to improve the results of surgery. The developed demonstrator is composed of two hyperspectral cameras covering a spectral range of 400–1700 nm. Furthermore, a hardware accelerator connected to a control unit is used to speed up the hyperspectral brain cancer detection algorithm to achieve processing during the time of surgery. A labeled dataset comprised of more than 300,000 spectral signatures is used as the training dataset for the supervised stage of the classification algorithm. In this preliminary study, thematic maps obtained from a validation database of seven hyperspectral images of in vivo brain tissue captured and processed during neurosurgical operations demonstrate that the system is able to discriminate between normal and tumor tissue in the brain. The results can be provided during the surgical procedure (~1 min), making it a practical system for neurosurgeons to use in the near future to improve excision and potentially improve patient outcomes.

## 1. Introduction

Currently, patients with brain cancer continue to have very poor survival rates. Surgery is one of the mainstays of treatment, together with radiotherapy and chemotherapy [1]. Brain tumors are classified based on their histology and molecular parameters [2]. Malignant gliomas are the most common form of primary brain tumors in adults and cause between 2 and 3% of cancer deaths worldwide [3]. Since brain tumors diffusely infiltrate into the surrounding normal brain tissue (especially gliomas), it is extremely difficult for the surgeon to accurately differentiate between tumor and normal brain tissue with the naked eye. In some cases, unintentionally leaving behind tumor tissue after the resection is unavoidable, and in other cases, too much normal brain tissue is resected in an effort to ensure complete excision. Over-resection can produce permanent neurological deficits that affect patient quality of life [4]. In contrast, several studies have demonstrated that tumor tissue left behind during surgery is a major cause of morbidity and mortality and represents the most common cause of tumor progression [5,6,7].

Several image guidance tools, such as intra-operative neuro-navigation, intra-operative magnetic resonance imaging (iMRI), and fluorescent tumor markers (for example 5-aminolevulinic acid, 5-ALA), have been commonly used to assist surgeons in the identification of brain tumor boundaries. However, these technologies have several limitations. One limitation is related to the brain shift phenomenon [8]. During craniotomy, the opening of the skull and dura inevitably leads to movement of the brain. This typically manifests as herniation of the brain into the craniotomy defect under pressure from the underlying tumor, or the slump of the brain due to drainage of cerebrospinal fluid and the administration of mannitol. Similarly, following resection of the tumor, the residual brain tissue may slump towards the surgical cavity. This brain deformation invalidates the patient-to-image mapping and reduces the effectiveness of using pre-operative images for intra-operative surgical guidance. Thus, neuronavigation systems relying on preoperative image data have decreasing accuracy as the surgical procedure progresses [9,10,11]. iMRI solves the problem of brain shift, mapping the tumor margins intra-operatively, but this method has poor spatial resolution and significantly extends the duration of the surgery, with a limited number of images that can be obtained [12]. Finally, although 5-ALA can identify the tumor boundaries, it produces relevant knock-on effects for the patient and can only be used for high-grade tumors [13,14]. Thus, there is no current device that helps in the accurate definition of brain tumor boundaries during surgical procedures. A label-free and non-ionizing imaging modality would be an ideal solution to this problem.

Hyperspectral imaging (HSI) is a non-contact, non-ionizing, and minimally invasive sensing technique that has been used in medical applications for more than two decades [15,16]. Unlike standard red, green, and blue (RGB) or multispectral images (which have a few more bands than the RGB image), hyperspectral (HS) images cover a wide range of the electromagnetic spectrum, and are able to capture a large number of contiguous and narrow spectral bands. This high amount of information conforms the spectral signature, which offers the possibility of distinguishing between each type of material or substance presented in the captured scene. HSI is an emerging imaging modality, and promising results have been shown with respect to cancer detection. Akbari et al. performed a study to identify gastric tumors in human ex vivo tissues, employing an HS system capable of capturing images ranging in size between 1000 and 250 nm [17]. From their experiments, they determined that the spectral regions between 1226 and 1251 nm and 1288 and 1370 nm are the most suitable ranges for distinguishing between non-cancerous and cancerous gastric tissue. Laryngeal cancer has been investigated by Regeling et al. using a flexible endoscopy coupled to an HSI system that is able to obtain HS cubes in the region between 390 and 680 nm [18]. Additionally, in this area Kester et al. developed a real-time snapshot HSI endoscope system based on an image mapping technique that is capable of operating at frames rates of 5.2 fps (frames per second), obtaining HS cubes in the range between 450 and 650 nm, with a spatial resolution of 100 µm [19]. In prostate cancer, Akbari et al. employed an HSI system to capture in vivo images (in the range between 450 and 950 nm) of mice affected by human prostate tumors [20]. Their results showed a maximum sensitivity of 92.8% and a specificity of 96.9% in the classification of malignant and non-malignant regions. Several studies have been carried out employing HSI for breast cancer diagnosis. Hou et al. developed a laser diode-induced hyperspectral system especially designed for breast cancer diagnosis, achieving higher accuracy and resolution as well as faster processing than other brain cancer diagnosis systems [21]. In addition, ex vivo breast cancer tissues were studied by Kim et al. to extract their regions of interest and thus differentiate between cancerous and non-cancerous tissues, employing a hyperspectral system that covered the region between 380 and 780 nm [22]. The same group also worked in the classification of these ex vivo breast cancer tissues using HSI, obtaining sensitivity and specificity of 98% and 99%, respectively [23]. In vivo colorectal tumors were also studied by Han et al. using a flexible hyperspectral colonoscopy system to discriminate between malignant colorectal tumors and normal mucosa in human patients [24]. Moreover, in vitro colon biopsy samples were analyzed by Masood et al. using a HSI system based on a tuned light source and a charge-coupled device (CCD) camera coupled to a microscope with 40× magnification (covering the range between 440 and 700 nm), obtaining accuracy results of 90% in the differentiation of benign and malignant patterns [25]. In vitro hyperspectral colon tissue images were also classified and segmented using morphological analysis and wavelet-based segmentation in [26,27]. HSI has also been applied to analyze skin cancer using visible-to-near-infrared (VNIR) information, obtaining promising results in the discrimination between melanoma and normal skin [28,29]. Other types of tumors have been also studied and analyzed using HSI, such as those of the head and neck [30], oral tissue [31], and tongue [32,33,34]. Nevertheless, HSI systems are not standardized, as different technologies were used in these studies. HS cameras generally use CCD sensors for VNIR applications (covering the range between 400 and 1000 nm) while indium gallium arsenide (InGaAs) sensors are used for near-infrared (NIR) applications (covering the range between 1000 and 1700 nm), since the quantum efficiency of the CCD sensors is quite low above 1000 nm. As a result, in some applications, more than a single camera is required to cover a broadband spectral range to study the suitable spectral range of the application, as is done in the creation of a spectral signature library for abdominal organs, arteries, and veins [35], or in the study of detection and analysis of intestinal ischemia during surgery [36]. The illumination systems used in HSI applications are mainly based on halogen or xenon lamps, and sometimes, optical fibers are used for light transmission, like in the diffuse reflectance spectroscopy used for early detection of malignant changes in the oral cavity [37].

The work presented in this paper was done as part of the HypErspectraL Imaging Cancer Detection (HELICoiD) project [38,39,40]. HELICoiD is a European Future and Emerging Technologies (FET) project with the goal of developing a demonstrator capable of discriminating between tumor and normal brain tissue, which can be used during neurosurgical operations. This demonstrator is designed to help surgeons with brain tumor resection, avoiding the excessive extraction of normal tissue and preventing small remnants of tumors from being left behind. Such precise delimitation of the tumors boundaries will improve the results of the surgery and is expected to improve patient outcomes. Although some parts of the system have been already described in previous works [41,42,43,44,45], in this paper we present, for the first time, a comprehensive description of the full system, including parts not previously addressed like the integration with hardware acceleration. We also present the measurements of the total times (for acquisition and processing), and the results using the complete training database and data from five new patients (which were not employed to train the classifier) to validate the overall system.

## 2. Materials and Methods

**Figure 1:**
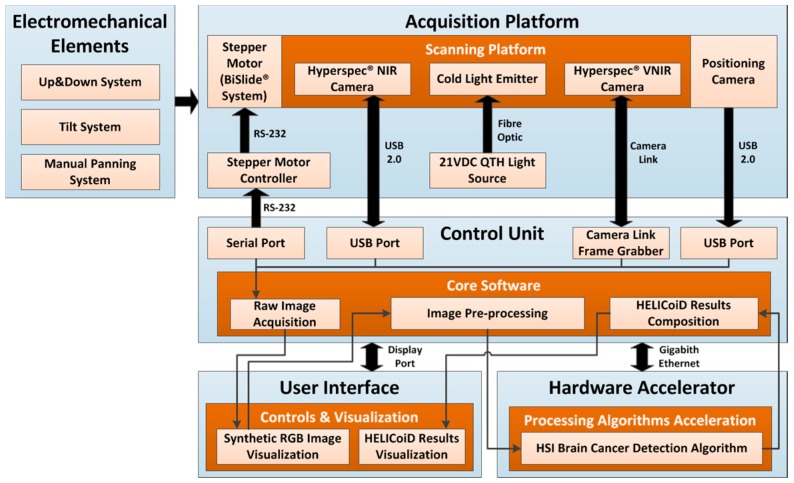
The HELICoiD demonstrator block diagram. QTH: Quartz Tungsten Halogen.

This section describes the HSI instrumentation developed for the detection of brain cancer intraoperatively. Figure 1 shows the block diagram of the demonstrator where all the parts of the system and their interconnections are presented. The acquisition platform is formed by two pushbroom HS cameras, covering the spectral range from 400 to 1700 nm, and the illumination system, mounted on a scanning platform guided by a high-precision stepper motor. The control unit is in charge of managing all the components of the system, while the hardware accelerator has the goal of speeding up the HS brain cancer detection algorithm in order to perform intraoperatively. The electromechanical elements allow the demonstrator’s operator to focus and obtain the image in optimal conditions. Finally, the user interface was developed in a user-friendly way, facilitating the use of the system by non-expert users. Each of these parts will be described in detail in the following sections.

### 2.1. Acquisition Platform

**Figure 2:**
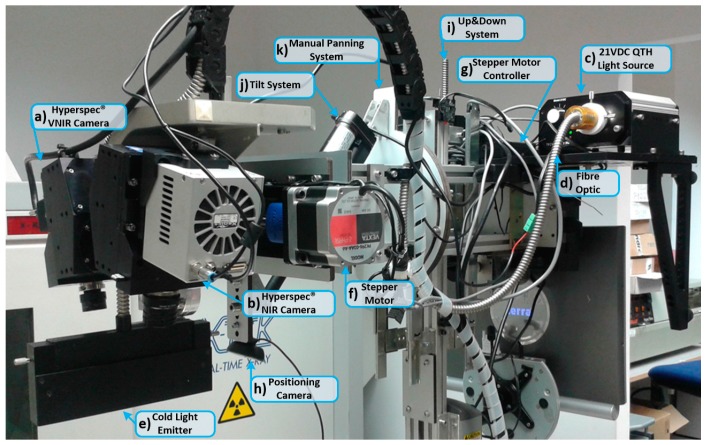
The HELICoiD demonstrator acquisition platform. (**a**,**b**) VNIR and NIR HS cameras mounted on the scanning platform; (**c**–**e**) QTH light source connected to the fiber optic system for the light transmission to obtain cold light emission in the scanning platform; (**f**,**g**) Stepper motor coupled to the spindle and connected to the stepper-motor controller to perform the linear movement of the cameras; (**h**) Positioning of the RGB camera used to identify the position of the cameras’ field of view (FOV); (**i**) The Up&Down system used to focus the HS cameras; (**j**) and (**k**) Tilt and manual panning systems employed to correctly orientate the scanning platform.

The acquisition platform locates all the elements required to capture the HS images (also called HS cubes). Two HS cameras that cover the spectral range from 400 to 1700 nm are employed. Using these two cameras, two different HS cubes are generated: one in the VNIR spectral range (from 400 to 1000 nm) and another one in the NIR spectral range (from 900 to 1700 nm). Four different elements compose the acquisition platform: the HS cameras, the scanning platform, the illumination system, and the positioning camera. Figure 2 summarizes all the elements that are placed in the acquisition platform of the demonstrator.

#### 2.1.1. Hyperspectral Cameras

HS cameras are mainly classified into four different types depending on the method employed to obtain the HS cube: whiskbroom (point-scanning) cameras, pushbroom (line-scanning) cameras, cameras based on spectral scanning (area-scanning or plane-scanning), and snapshot (single shot) cameras [46]. The HS cameras selected for the acquisition platform of the system are the Hyperspec^®^ VNIR A-Series (Figure 2a) and the Hyperspec^®^ NIR 100/U (Figure 2b) cameras, manufactured by Headwall Photonics Inc. (Fitchburg, MA, USA). These HS cameras are based on a line-scanning technique. The camera sensor is a two-dimensional detector array in which one of the spatial dimensions and the complete spectral dimension of the scene are captured in one single shot (called a frame). The second spatial dimension is obtained by shifting the camera’s field of view (FOV) relative to the scene by means of a linear motion system. These cameras offer the best compromise between spectral and spatial resolution and acquisition time. The spectral range covered by both cameras is between 400 and 1700 nm (VNIR and NIR). This range has been selected with the aim of finding the most relevant spectral regions where the tumor and normal brain tissues can be distinguished using machine learning algorithms. The main characteristics of the selected cameras are as follows:

- The Hyperspec^®^ VNIR A-Series model covers spectral range from 400 to 1000 nm. It has a dispersion per pixel of 0.74 nm and a spectral resolution of 2–3 nm (with a 25-μm slit), and is able to capture 826 spectral bands and 1004 spatial pixels. This device integrates a silicon CCD detector array (Adimec 1000-m, Adimec Electronic Imaging, Inc., Woburn, MA, USA) with a minimum frame rate of 90 fps. This sensor is a monochromatic camera connected to the control unit using a PIXCI^®^ Camera Link Interface (EPIX, Inc., Buffalo Grove, IL), which provides a data transmission rate up to 255 MB/s. The lens used in this camera is a Xenoplan 1.4 (Schneider Optics, Hauppauge, NY, USA) with a focal length of 22.5 mm and a broadband coating for the spectral range of 400 to 1000 nm.
- The Hyperspec^®^ NIR 100/U model covers the spectral range from 900 to 1700 nm. It has a dispersion per pixel of 4.8 nm and a spectral resolution of 5 nm (with a 25-μm slit), being able to capture 172 spectral channels and 320 spatial pixels. This system incorporates an indium gallium arsenide (InGaAs) detector array (Xeneth XEVA 5052, Xenics nv, Leuven, Belgium), which provides a fast response, high quantum efficiency, and low dark current for the sensor area. This system has a frame rate of up to 100 fps. This camera is connected to the control unit by a USB 2.0 interface with a transfer rate up to 60 MB/s. The lens used with this camera is a Kowa LM25HC-SW 1.4 (Kowa Optimed Deutschland GmbH, Düsseldorf, Germany) with 25 mm of focal length and a broadband coating for the spectral range of 800–2000 nm.

#### 2.1.2. Illumination System

HS cameras require strong and precise illumination of the scene to be captured in order to avoid external interferences produced by the environmental illumination where the capture is being performed. The illumination system used in this demonstrator is based on a quartz tungsten halogen (QTH) lamp of 150 W with a broadband emission between 400 and 2200 nm. This type of lamp is suitable for HS applications due to the high homogeneity of its spectrum across the entire spectral range [47]. The light source where the lamp is installed is a TechniQuip’s Model 21 DC source light (TechniQuip, Pleasanton, CA, USA) connected to an optical fiber that transmits the light to a cold light emitter, ending in double glass isolation with an air chamber in the middle. Using this cold light system, the high temperature produced by the QTH lamp is isolated from the brain surface, since a high temperature irradiating over the brain surface can cause damage and even premature cell death [48]. Figure 2c shows the light source placed in the back of the system connected to the optical fiber (Figure 2d) that transmits the light to the cold light emitter located in the scanning platform (Figure 2e).

Although the illumination system employed in this demonstrator is able to avoid the interference of environmental illumination, HSI requires calibration of the raw images to be performed for correct processing of the data. In the calibration process, the significant signal variations caused by the non-uniform illumination over the surface of the captured scene are corrected. The acquired raw image is calibrated using white and dark reference images. These reference images are acquired by the system with the VNIR and NIR cameras separately, but in the same illumination conditions inside the operating theatre before the start of the operation. A white reference image is acquired from a Spectralon^®^ tile (SphereOptics GmbH, Herrsching, Germany), a type of material that reflects the 99% of the incoming radiation in the full spectral range considered in this work. This white reference is placed at the same location where the patient’s head will be placed during the surgery, thus taking into account all the real light contributions. The dark reference image is obtained by keeping the camera shutter closed and is used to avoid the dark currents produced by the camera sensor. The HS-calibrated image is calculated by Equation (1), where β is the calibrated image, α is the raw image, and γ and δ are the white and dark reference images, respectively:

(1) $$\beta =100\cdot \frac{\alpha -\delta }{\gamma -\delta }$$

**Figure 3:**
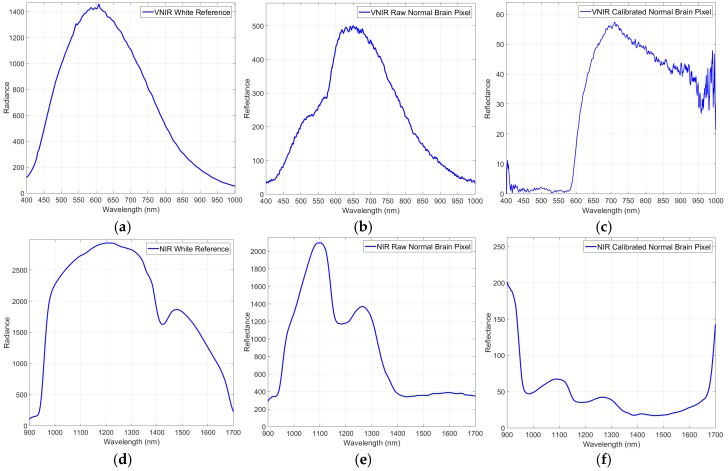
Calibration process of a certain pixel of the VNIR and NIR cameras. (**a**) The VNIR white reference spectrum; (**b**,**c**) The VNIR raw and calibrated spectra of a pixel of normal brain tissue; (**d**) The NIR white reference spectrum; (**e**,**f**) The NIR raw and calibrated spectra of a pixel of normal brain tissue.

Figure 3a shows the white reference tile spectrum obtained with the VNIR camera, while Figure 3b,c respectively present raw and calibrated spectrum examples of normal brain tissue pixels. In Figure 3d the representation of the white reference tile spectrum obtained with the NIR camera can be seen, and in Figure 3e,f, the raw and calibrated spectra of a normal brain tissue pixel are shown. Based on the repeatability experiments performed with the system and taking into account that the white reference tile is used only a few minutes for the calibration, through measurements it is confirmed that the spectrum of the certified white reference tile does not show perceptible changes over time.

#### 2.1.3. Scanning Platform

Commonly, in the HS found in laboratories based on pushbroom cameras, the camera is usually fixed and the sample to be captured is moved, although some few examples can be found of moving cameras [49]. In brain tumor applications, it is not possible to move the brain of the patient to perform the capture; instead, the HS cameras (Figure 2a,b) are installed in a scanning platform together with a cold light emitter (Figure 2e). The scanning platform provides the necessary movement for the pushbroom scanning. This scanning platform is composed of a spindle and a stepper motor, called the BiSlide^®^ motor-driven assembly (Velmex, Inc. Bloomfield, NY, USA, Figure 2f). The spindle has a size of 1 m and allows the cameras to capture a scene of a maximum size of 230 mm in the X-axis. The step resolution of the scanning platform is 6.17 µm. The stepper motor is managed by a Velmex VXM^®^ stepping motor controller (Velmex, Inc. Bloomfield, NY, USA, Figure 2g). This motor controller is connected to the control unit via a serial protocol and its programming is accomplished through a Recommended Standard 232 (RS-232) protocol.

#### 2.1.4. Positioning Camera

The positioning camera is installed in the acquisition platform to visualize the area that will be captured by the HS cameras. Since every HS camera sensor captures only one spatial line of the scene, it is not possible to determine the exact position of the current pushbroom frame over the brain. For this reason, the inclusion of an additional standard RGB camera in the acquisition platform was required, correctly aligned with the FOV of the HS cameras, in order to identify the area of the brain surface to be captured. However, unlike the HS cameras, this positioning camera is placed in a fixed position. This camera permits the user to visualize the complete area that is going to be captured by the cameras, allowing the system to be easily positioned in the correct place. Figure 2h shows the positioning camera placed in the acquisition platform below the scanning platform.

#### 2.1.5. Electromechanical Elements

Three different electromechanical elements were installed in the HS acquisition system. These elements provide several degrees of freedom to the system, which are required to focus and orientate the cameras in a convenient way for obtaining high quality images. The Up&Down system (Figure 2i) allows the movement of the acquisition platform in the Y-axis to focus the camera. Keeping the HS images well focused is fundamental for obtaining good quality spectral signatures. Effectively, the spectral signature of each pixel is distorted in the case they are unfocused. The focus of the system is performed by looking an X-Lambda image (all the bands of the captured line in a spatial 2D image) captured by the sensor, where the lambda is the wavelength. The focusing distance between the exposed brain tissue and the lens of the cameras is 40 cm. This distance is determined by the distribution of the HS cameras in the scanning platform. The FOV of both cameras is oriented and aligned to the beam of the cold light emitter to obtain the highest reflectance value in the sensor. Furthermore, this distance is determined by the minimum security distance (30 cm) that must exist between the exposed brain and the nearest element of the demonstrator (in this case, the cold light emitter). The Up&Down system is composed of a 24-VDC motor coupled to a spindle, allowing a displacement of ±7.75 cm. On the other hand, the tilt system (Figure 2j) is composed of a 12-VDC linear actuator that permits the rotation of the scanning platform 40° forward and backward. Finally, the manual panning system (Figure 2k) is employed to manually rotate (up to 45° to the left and 45° to the right) the scanning platform, using an aluminum plate.

### 2.2. Control Unit

The control unit (CU) is responsible for managing all the subsystems that comprise the demonstrator. This CU is a computer based on an Intel^®^ Core™ i7-4770k 3.5 GHz quad-core processor, with 8 GB of Random Access Memory (RAM) and a high-capacity 512 GB solid-state drive with write speeds exceeding 500 MB/s. Specific software was developed to manage and integrate the different elements that conform the acquisition platform, allowing the user to perform the HS image acquisition in an easy and effective way. Furthermore, the CU is in charge of executing the HS brain cancer detection algorithm together with the hardware accelerator in order to finally present the tumor boundary prediction.

#### HS Image Acquisition Software

Customized software for image acquisition was developed due to the need to automate and accelerate the capture of both HS cameras of the system. The simplification of the acquisition procedure ensures easy interaction of the user with the system as well as reduced time needed to capture the HS images during neurosurgical procedures.

**Figure 4:**
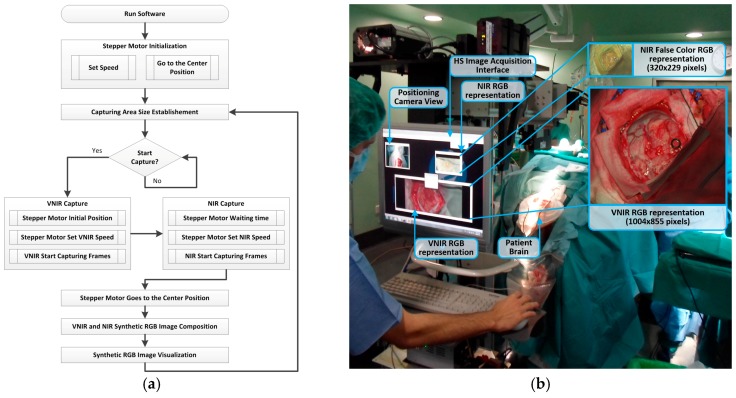
(**a**) HS image acquisition software flow diagram; (**b**) HS image acquisition user interface (and the RGB representations of each HS cube) being used during a neurosurgical intervention at the University Hospital Doctor Negrin of Las Palmas de Gran Canaria (Spain).

To develop this software, three different software development kits (SDKs) were integrated, belonging to the two HS cameras and the stepper motor controller. Figure 4a shows the HS image acquisition software flow diagram for the capturing procedure. Firstly, after running the program, the scanning platform is initialized, detecting and establishing the absolute zero of the motor position. Then, the platform is positioned at the center of the scanning area. Taking into account the x-size value of the capturing area established by the user through the graphical user interface (GUI), the scanning platform is moved to the initial position. The VNIR capturing process is performed starting from the right to the left of the platform with the stepper motor speed fixed to 3 mm/s. This speed is calculated according to the pixel size (0.1287 mm and 0.48 mm for the VNIR and NIR cameras, respectively) and the frame rate of the camera (90 fps and 100 fps for the VNIR and NIR cameras, respectively). When the VNIR capture is done, the stepper motor stops at the final position, waits a few milliseconds to stabilize the system structure, and fixes the speed to 5 mm/s. Then, the NIR capturing process begins. This capture is performed starting from the left to the right of the platform. After that, the stepper motor moves the scanning platform to the central position. Then, the synthetic RGB images of both HS cubes are generated by selecting three bands that correspond with red (708.97 nm), green (539.44 nm), and blue (479.06 nm) colors for the VNIR image, and three bands of the NIR cube to generate a false color RGB image (red: 1094.89 nm, green: 1247.44 nm and blue: 1595.45 nm). These bands are selected to maintain the compatibility with the original software (Hyperspec^®^ III software, Headwall Photonics Inc., Fitchburg, MA, USA) provided by the camera manufacturer. Using this technique for the acquisition process, a speedup of 3× with respect to the original software is achieved. The maximum image size provided by the system is 1004 × 1787 pixels (129 × 230 mm) for the VNIR image, and 320 × 479 pixels (153 × 230 mm) for the NIR image, with spatial resolutions of 128.7 µm and 480 µm, respectively.

Figure 4b shows the acquisition system being used during a neurosurgical operation and the RGB synthetic images of the captured HS cubes (VNIR and NIR) where their image sizes and relative spatial resolutions can be seen. The time employed by the system to obtain the maximum size image using the manufacturer’s software is ~240 s for the VNIR image and ~140 s for the NIR image. However, employing the acquisition software developed in this work, the acquisition time for the maximum image size is reduced to ~80 s and ~40 s for the VNIR and NIR cameras, respectively.

### 2.3. Hardware Accelerator

Due to the high computational cost of the developed HS brain cancer detection algorithm and the large amount of data generated by the HS cameras, it is necessary to use a hardware accelerator (HA) where the most time-consuming parts of the algorithm are implemented. Therefore, the algorithm must be highly parallelized for processing to be completed during neurosurgical operations.

The HA selected for this purpose is the Kalray Massively Parallel Processor Array (MPPA^®^) EMB01 board (Kalray S.A., Montbonnot Saint Martin, France) with a multiple instruction, multiple data (MIMD) many-core processor [50]. This accelerator is focused on computationally-intensive low-power embedded applications. The MPPA^®^ EMB01 processing performance reaches 230 GFlops, which, for the 5-W power consumption reported, turns into 46 GFlops/W, a much higher figure compared to other kinds of high-performance platforms.

**Figure 5:**
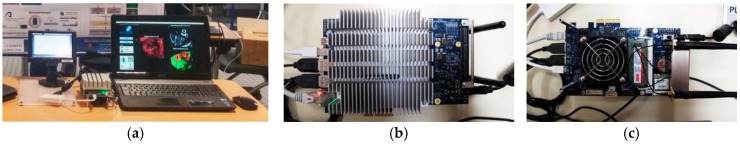
The Kalray MPPA^®^ EMB01 Platform: (**a**) Developer environment; (**b**) EMB01 top view where the host module is located; (**c**) EMB01 bottom view where the carrier board is placed.

The MPPA^®^ EMB01 board contains a standard host ×86 ComExpress module working as an embedded computer, and a carrier board containing the MPPA-256 many-core chip. Figure 5a shows the MPPA^®^ board (in the center of the image) connected to a preliminary environment developed to execute the hardware accelerated part of the algorithm. The host module side of the board (Figure 5b) is composed of an AMD G-T40E Dual Core Processor with an integrated graphics processor unit (GPU) running a CentOS 7 GNU/Linux operative system (OS) instance with 4 GB of RAM, 1 Peripheral Component Interconnect Express (PCIe) Gen2×2 for communication with the MPPA^®^-256 many-core chip, and a 16-GB solid-state drive (SSD) as a system disk. The carrier board can be seen in Figure 5c. It features an MPPA^®^-256 many-core processor (under the fan). It also contains 4 GB of RAM and 64 MB of flash memory plus the host PCIe Gen2×2 port to communicate with the dual core processor.

The Kalray MPPA-256 is a single-chip many-core processor that assembles 256 user cores distributed in 16 clusters running at 400 MHz. This chip comprises 256 user cores—32-bit very long instruction word (VLIW) processors with floating point units—distributed in several computing clusters. Additionally, this platform contains quad-core input/output (I/O) subsystems to manage the communications with the clusters. A network-on-a-chip (NoC) manages the synchronization and communications among the compute clusters and the I/O subsystem. Each cluster gathers 2 MB of memory—which is shared among the 16 cores—as well as a resource management (RM) core aimed at running the cluster operating system (NodeOS) and managing events and interrupts, and a direct memory access (DMA) module to transfer data from the shared memory to the NoC and vice versa. This architecture presents two main advantages: first, the system parallelization complexity is maintained within reasonable limits as the MPPA^®^ includes mechanisms such as POSIX (Portable Operating System Interface), OpenMP, and OpenCL; and secondly, in comparison with other architectures like GPUs or field programmable gate arrays (FPGAs), the MPPA^®^ platform leads in terms of energy efficiency [51].

### 2.4. HS Training Database

Employing the HELICoiD demonstrator, a total of 36 HS cubes of in vivo brain tissue belonging to 22 different patients were acquired from two different hospitals (the University Hospital Doctor Negrin at Las Palmas de Gran Canaria, Spain, and the University Hospital of Southampton, Hampshire, UK) in two data acquisition campaigns. The study protocol and consent procedures were approved by the Comité Ético de Investigación Clínica-Comité de Ética en la Investigación (CEIC/CEI) for the University Hospital Doctor Negrin, and the National Research Ethics Service (NRES) Committee South Central–Oxford C for the University Hospital of Southampton. Written informed consent was obtained from all subjects.

**Figure 6:**
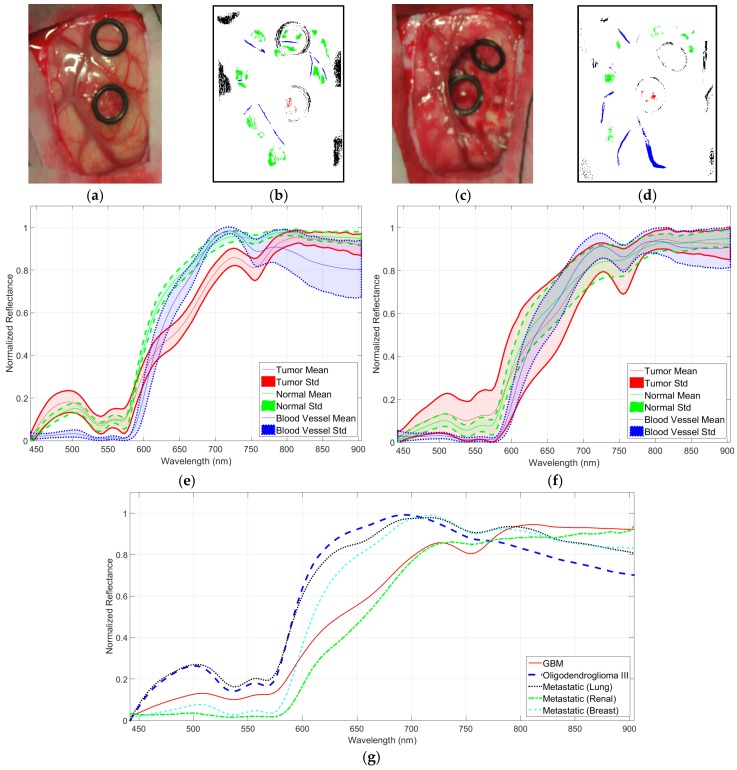
(**a**) Synthetic RGB representation of a VNIR HS cube of the a patient’s brain surface affected by a metastatic breast carcinoma (bottom marker) identified before resection started and (**b**) the training map where normal tissue, tumor tissue, blood vessels/hypervascularized tissue, and background were labeled using green, red, blue, and black colors, respectively; (**c**,**d**) Synthetic RGB representation and training map of the same patient but in an advanced stage of the tumor resection; (**e**) Mean and standard deviation of the pre-processed labeled spectral signatures of one patient affected by a GBM tumor (red), with labeled normal tissue pixels (green), and labeled blood vessels/hypervascularized tissue pixels (blue); (**f**) Mean and standard deviation of the pre-processed labeled spectral signatures of 13 patients affected by GBM tumors (with the same color identification); (**g**) Mean values of the pre-processed labeled spectral signatures of each type of tumor available in the training dataset.

The creation of the training dataset (the gold standard employed to train the HS brain cancer detection classifier) was performed in the following way. Firstly, after performing the craniotomy and durotomy, the operating surgeons placed some sterilized rubber ring markers over the brain surface areas that they considered with relative certainty to be made up of tumor or normal tissue, using the information provided by an image-guided navigation system based on preoperative computed tomography (CT) or magnetic resonance imaging (MRI), as well as macroscopic appearance. In the cases where the tumor area was superficial, markers were placed on the brain surface before the resection started. Figure 6a shows an example of the synthetic RGB representation of a captured HS cube where the markers were used to identify the normal tissue (top marker) and the tumor tissue (bottom marker) affected by metastatic breast carcinoma. In the cases where the tumor was in a deeper layer with respect to the normal tissue and it was clearly identified, no markers were used and the operating surgeon identified the tumor and healthy area immediately after the operation using the synthetic RGB image. After marker placement, the operator of the system captured an HS image. Depending on the location of the tumor, the images were acquired immediately after the dura removal (Figure 6a) when the tumor was superficially located, or in an advanced stage of the tumor resection (Figure 6c) when the tumor was deep-seated. Glioblastoma (GBM) heterogeneity is one of the main problems in establishing a gold standard for a training and validation dataset. For this reason, when possible, several images were captured at different stages of the operation of both of the necrotic core and the enhanced rim of the tumor tissue. Once the HS image was obtained, the operating surgeon performed a biopsy of the tissue located within the tumor tissue marker or within the clearly identified tumor area. The resected tissue was sent to the pathologist to confirm the presence or absence of tumor, and to specify its histopathological diagnosis (grade and type of tumor). The average size of the resected tumor sample obtained for pathological analysis was 0.5 × 0.5 mm, with a 0.2-mm depth, since HSI technique cannot practically penetrate into the tissue (in the case of NIR, the depth was of 1 mm at most). Normal tissue markers were only used as a reference for the labeling process carried out after the completion of the operation. It is not ethical to biopsy what is known to be normal brain tissue, as this can result in damage to the patient. In this preliminary study, the spectral differences between grey matter and white matter in normal brain sample were not taken into account. These differences were not relevant in this study as the intention was to only resect tumor tissue. The labeling of the HS cubes was performed using histopathological information (from the tumor tissue samples) and the knowledge of the operating surgeon (from the normal tissue samples) to create a training dataset for the supervised classifier of the HS brain cancer detection algorithm.

In order to increase the training dataset, a methodology for extracting the gold standard information from the HS cubes, based on the spectral angle mapper (SAM) algorithm [52], was developed and designed using Matlab^®^ GUIDE application. This SAM algorithm is an automated method for comparing the spectra of the pixels of a HS image with a well-known spectrum obtained from a reference pixel. The tool was employed by the corresponding operating surgeon after the completion of the operation to create the gold standard map for each captured HS image. Four different classes were established in this study: normal tissue, tumor tissue, blood vessel/hypervascularized tissue, and background (i.e., other materials or substances that can be presented in the surgical scenario but are not relevant for the tumor resection procedure). Therefore, normal class involves both grey matter and white matter tissue. The procedure to generate the neurosurgeon’s gold standard map is as follows. The user (usually the operating surgeon) loads the HS cube and selects a reference pixel, looking the synthetic RGB image at the location where a biopsy is done (where the tumor marker is placed) or at a location far enough from the tumor margins where the surgeon can be quite confident that the tissue is abnormal (in the case of tumor labeling). In the case of normal tissue, blood vessel/hypervascularized tissue, and background classes, the labeling is performed by selecting a reference pixel by the naked eye based on the surgeon’s knowledge and experience. Then, the most similar pixels to the selected reference pixel are highlighted, computed by using the SAM measurement, and the user configures the threshold that varies the tolerances on the selected pixels. Once the user considers that only the pixels belonging to one class have been highlighted, the selected pixels are assigned to that class. Neurosurgeons are instructed to select only a few sets of very reliable pixels instead of a wider set of uncertain pixels. Figure 6b,d shows an example of a gold standard map, where the labeled pixels that belong to tumor tissue, normal tissue, blood vessels/hypervascularized tissue, and background are identified with red, green, blue, and black colors, respectively.

In the end, the reliability of the training dataset is guaranteed by the use of (a) intraoperative MRI neuronavigation for locating tumor tissue; (b) the operating surgeon’s knowledge and experience in the labeling of normal tissues, blood vessels/hypervascularized tissues, and background samples; and (c) the pathological analysis of the resected tissues for the tumor labeling.

After a preliminary analysis of the spectral signatures of both HS cubes (VNIR and NIR), only the VNIR images were labeled and used to generate the training dataset for the brain cancer detection algorithm. This was because of the practical impossibility of performing reliable labeling of the NIR images due to the low spatial resolution of these HS cubes (Figure 4b). Figure 6e,f show the mean and standard deviations of the VNIR spectral signatures of normal brain tissue (green color), blood vessels/hypervascularized tissue (blue color), and tumor tissue (red color) affected by GBM. In Figure 6e, the intra-patient variability (of one patient affected by GBM) of the spectral signatures can be seen, while in Figure 6f, the inter-patient variability (of 13 patients affected by GBM) is shown. In these cases, the tumor samples were obtained from the center of the tumor in the brain surface identified using the intraoperative neuronavigation system. These spectral signatures were extracted from the VNIR HS cube after the application of the pre-processing chain of the HS brain cancer detection algorithm (described in the next section). Figure 6g shows the average spectral signatures of each tumor type comprising the training database. As can be seen in this figure, there are significant spectral differences between these types of tumors. However, this study has mainly addressed the discrimination between tumor tissue (involving all the types of tumors) and normal tissue.

Table 1 details the total number of pixels labeled per each class and type of tissue. The tumor class involves two different primary tumors (GBM and grade III anaplastic oligodendroglioma) and three different secondary tumors, also called metastatic tumors (lung, renal, and breast carcinomas). After labeling all the available data, a total of 377,556 spectral signatures were obtained from the training database. Using this training dataset, the supervised classification stage of the HS brain cancer detection algorithm was trained in order to generate the classification maps from a new patient during the surgical procedure. Although different types of tumors were included in the training database, for this preliminary study only one multiclass supervised classifier was generated to differentiate primarily between tumor and normal tissue. Only one classifier was used instead of a different one per each type of tissue due to the reduced number of samples obtained for each type of tumor.

**Figure 7:**
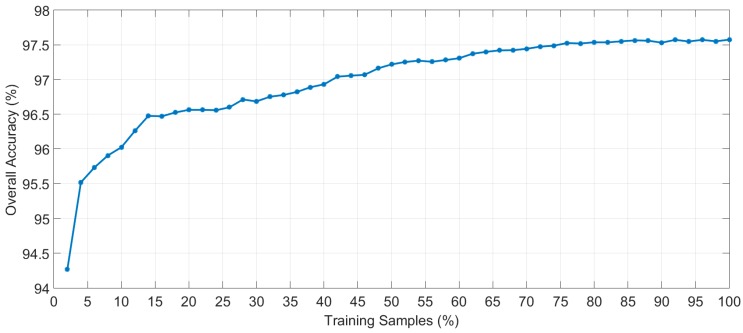
Overall accuracy evolution depending on the percentage of training samples employed to generate the supervised classification model of the HS brain cancer detection algorithm.

In order to determine the suitable percentage of samples of the training database that should be used to train the supervised algorithm, several experiments to generate and evaluate the supervised model were carried out employing different number of training samples. Each experiment was performed following a 10-fold cross-validation method to calculate the average overall accuracy result. Figure 7 shows the overall accuracy results varying the percentage of training samples with increments of 2%, starting at 2% and finishing at 100%. The evolution of the overall accuracy shows that when more than 75% of the training samples are used, the results stabilize, with overall accuracy of around 97.5%. With this experiment, it can be seen that there is no overfitting effect and the use of all the training samples will provide the best classification map.

### 2.5. Brain Cancer Detection Algorithm Implementation

The HS brain cancer detection algorithm developed in this research work aims to exploit both the spatial and spectral features of the HS images. The whole algorithm can be divided into two main steps: the off-line process and the in situ process. The off-line process is the part of the algorithm in which the information previously provided by the experts in labeled samples is employed to train the supervised stage of the algorithm. On the other hand, the in situ process is carried out during surgery inside the operating theatre when a new HS image is acquired from the undergoing patient. This part of the algorithm is implemented and accelerated using the HELICoiD demonstrator.

In summary, the in situ process is based on five main steps. Firstly, a new hypercube is acquired during a surgical procedure. Secondly, a pre-processing chain is applied to homogenize the spectral signatures of the HS cube. Thirdly, a supervised pixel-wise classification is performed in order to obtain a classification map, where different types of tissues are identified according to the information previously provided by medical doctors. The supervised classifier employed is the support vector machine (SVM) algorithm [53], previously trained in the off-line process with the HS training dataset. Fourthly, once the supervised classification map is obtained, a spatial–spectral homogenization is accomplished using k-nearest neighbors (k-NN) filtering, where a one-band representation of the hypercube is employed. The dimensionality reduction algorithm used to obtain the one-band representation of the hypercube is the principal component analysis (PCA) algorithm [45]. Finally, in order to obtain the definitive classification map (also called the HELICoiD three maximum density (TMD) map), the spatial–spectral supervised classification map is fused with a segmentation map, obtained via unsupervised learning, employing hierarchical K-means (HKM) clustering. The algorithm used to fuse both images is based on a majority voting (MV) approach.

**Figure 8:**
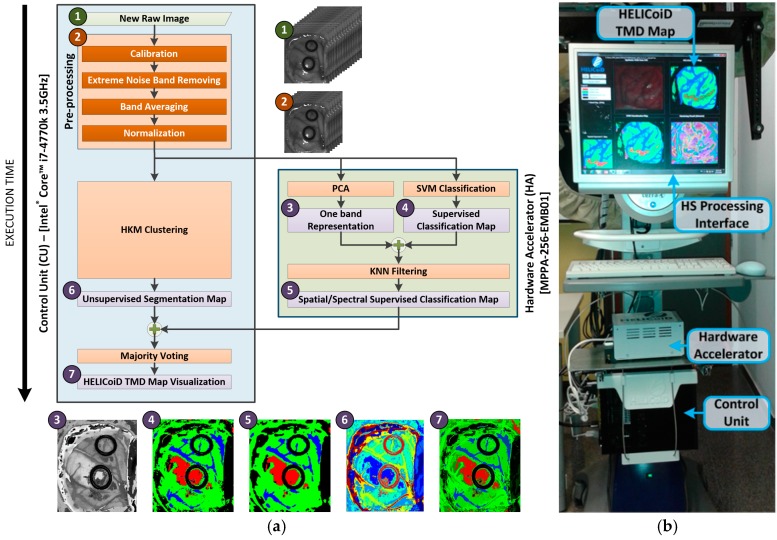
(**a**) HS brain cancer detection algorithm implementation flow diagram and the RGB representation of the output of each step; (**b**) Different parts related to the HS data processing of the HELICoiD demonstrator.

Figure 8a depicts the different blocks of the HS brain cancer detection algorithm, as well as their distribution in the implementation onto both platforms and the execution scheduling. Furthermore, the RGB representation of the outputs obtained at each step of the algorithm is also shown. The blue block represents the steps of the algorithm that were mapped to the CU, while the green block represents the steps mapped to the HA. As can be observed, the pre-processing stage, the HKM clustering and the MV algorithm are executed on the CU. In contrast, the spatial–spectral supervised classification stage, where the PCA, the SVM classification, and the KNN filtering are performed, is executed on the HA due to its high computational load.

The data flow sequence of the implementation follows the next steps. Firstly, the raw image is pre-processed on the CU and the resulting HS cube is sent to the HA through the Gigabit Ethernet interface, to be employed as the input of the PCA and SVM classification algorithms. The same HS cube is used in the CU as the input of the HKM clustering algorithm. Secondly, HKM clustering is executed on the CU, while the spatial–spectral supervised classification—PCA, SVM classification and KNN filtering—is executed on the HA. Both the unsupervised and the supervised stages are executed simultaneously. In addition, the PCA algorithm and the SVM classification are executed in parallel in the HA. Finally, once the previous stages are finalized, the MV algorithm is executed on the CU to compute the final HELICoiD TMD map. This TMD map is a RGB representation of the first three major probabilities per cluster obtained from the HKM clustering algorithm, where the brain tumor is marked in red. This image is shown to the user (the neurosurgeon) through the HS processing interface. Figure 8b shows the different parts that comprise the HELICoiD demonstrator in relation to HS data processing.

## 3. Experimental Results and Discussion

The validation of the HELICoiD demonstrator was performed during neurosurgical operations at the University Hospital Doctor Negrin of Las Palmas de Gran Canaria, employing the data of four different patients affected by different types of tumors. Table 2 details the characteristics of the validation database used to test the developed HS brain cancer detection system and the corresponding pathological diagnosis. Seven different images were included. These images involved normal brain tissue acquired during the first stage of the surgical operation, used to test if the system included false positives when no tumor as present in the image, and three different types of primary tumors.

**Figure 9:**
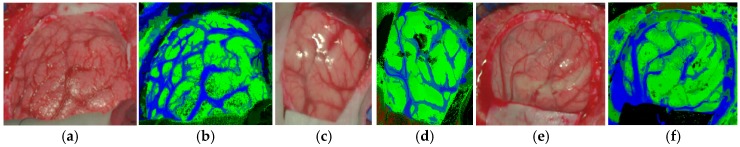
Normal brain image results obtained from the validation database employing the HELICoiD demonstrator: (**a**,**b**) synthetic RGB image and TMD map of the P1C1 HS image; (**c**,**d**) synthetic RGB image and TMD map of the P2C1 HS image; (**e**,**f**) synthetic RGB image and TMD map of the P3C1 HS image.

**Figure 10:**
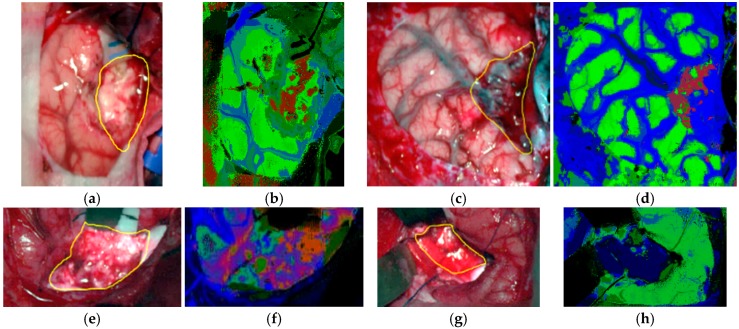
Tumor tissue identification results obtained from the validation database employing the HELICoiD demonstrator: (**a**,**b**) synthetic RGB image and TMD map of the P2C2 HS image; (**c**,**d**) synthetic RGB image and TMD map of the P4C1 HS image; (**e**,**f**) synthetic RGB image and TMD map of the P1C2 HS image; (**g**,**h**) synthetic RGB image and TMD map of the P3C2 HS image.

The TMD maps of the validation database obtained by the HELICoiD demonstrator during the surgical operations and their respective synthetic RGB images are shown in Figure 9 and Figure 10. The TMD maps are represented in four colors that can be mixed depending on the density of each class presented in the image. Figure 9 shows the results obtained from the normal brain images. In these results, it can be seen that the system does not present any false positives in the parenchymal area, and normal tissue and blood vessels are clearly identified. Furthermore, bright pixels, which can be found in the images due to the light reflections over the arachnoid of the brain or due to the presence of surgical serum in the surface, are identified as background pixels. On the other hand, Figure 10 shows the results obtained from the HS images of the brain surface affected by a tumor, where the tumor areas are surrounded with a yellow line in the synthetic RGB representations. These results offer a clear indication that the HELICoiD demonstrator is able to identify the tumor tissue presented in the images. In Figure 10b, there are some false positives in the bottom corner of the TMD map, however, this false information is located outside the area of exposed brain parenchyma and thus, it does not affect the neurosurgeon decisions during the tumor resection. It is worth noting that two of the cases (Figure 10b,d) identify two tumor types (grade II oligodendroglioma and grade I meningioma) for which there are no spectral signatures within the training database. These results highlight the robustness and the generalization capabilities of the HELICoiD demonstrator to identify other types of tumor rather than only the ones available in the HS training database. Finally, it should be mentioned that the tumor identification becomes more difficult when the tumor is located deeper in the brain. Figure 10f,h show the TMD maps of GBM tumors at an advanced stage of the surgical procedure. It can be seen that, in case of Figure 10f, the tumor tissue is clearly identified although it is located in a deep layer. However, in Figure 10h, there is no correct identification of the tumor tissue due to problems with shadows and the presence of extravasated blood in the tumor area. Since HSI is not able to penetrate into the surface, extravasated blood present in the image is identified as a hypervascularized tissue class (blue color) in the TMD map.

Table 3 shows the execution times obtained using the HELICoiD demonstrator to acquire and process the validation database during surgery. To assess the processing times obtained using the hardware acceleration in the spatial-spectral supervised classification stage, Table 3 also shows the processing times obtained when the whole algorithm is implemented in the CPU, i.e., sequential time results. The total processing time required in the accelerated version is computed taking into account the maximum time obtained between the spatial–spectral supervised classification (PCA + SVM + KNN) and the unsupervised clustering (HKM). In summary, when the hardware accelerator is not employed, the spatial–spectral supervised classification is the most time-consuming stage. In contrast, an average speedup factor of 24× is achieved in the spatial–spectral supervised classification stage when the hardware accelerator is employed, becoming the unsupervised clustering the limiting factor in this case. These results show that the proposed system provides a TMD map of the captured scene during the surgery in approximately 1 min, depending on the size of the captured image.

## 4. Conclusions

In this study, a novel visualization system based on HSI was developed to aid surgeons in the difficult task of identifying brain tumor boundaries during neurosurgical procedures. The identification of tumor boundaries and tumor infiltration into normal brain tissue is extremely important in order to avoid excessive resection of normal brain tissue and to avoid unintentionally leaving behind residual tumor. Using only RGB information, the naked eye cannot be used to accurately determine the boundaries of the tumor, especially in gliomas where tumor heterogeneity is extremely high. In addition, intraoperative neuronavigation based on CT and MRI is problematic due to brain shift, producing a significant error between the real position of the tumor boundaries respect to the CT or MRI information. As a proof-of-concept, the demonstrator developed in this study was able to generate thematic maps of the exposed brain surface using spectral information of the VNIR range (between 400 and 1000 nm). These thematic maps differentiate between four different classes: normal tissue, tumor tissue, blood vessels/hypervascularized tissue, and background. In these maps, the tumor boundaries can be easily identifiable. Only the information obtained from the VNIR camera has been employed to generate the gold standard for the training of the classification algorithm and validate its results. Due to the low spatial resolution of the NIR camera, it is not possible to perform reliable labeling of the NIR HS cubes. Although some preliminary analysis of the NIR images performed by the research team reveal that the use of the NIR spectral range could help in the identification of blood vessels and extravasated blood, NIR images alone are not relevant for the goal of this study. An HS brain cancer detection algorithm, based on unsupervised and supervised machine learning approaches, was developed and implemented in the system. The supervised algorithm was trained by employing a labeled dataset composed of more than 300,000 spectral signatures, extracted by medical doctors from 36 different HS cubes captured with the acquisition system from 22 different patients from Spain and UK. In this preliminary study, only one multiclass classifier was generated for the supervised part of the algorithm, employing all the types of tumors available in the training database to distinguish mainly between tumor and normal tissue, without identifying the different types of tumors. The implementation of the algorithm was partitioned between the control unit and a hardware accelerator, where the higher computational tasks were implemented in a many-core platform to achieve intraoperative processing (~1 min). The demonstrator was validated using seven HS images obtained in four neurosurgical operations. The TMD maps demonstrate that the system did not introduce false positives in the parenchymal area when no tumor was present and it was able to identify different types of tumor that were not present in the training database. Currently, further investigations are being carried out by the research team in order to enlarge the training database and the validation database with more patients and types of tumors. Additionally, the fusion of both types of HS images (VNIR and NIR) is being investigated in order to investigate if the NIR information could help to more accurately distinguish the boundaries between the tumor tissue and the surrounding hypervascularized normal tissue. Furthermore, an extensive clinical validation of the system must be carried out. In this clinical validation, a comprehensive pathological analysis of the entire tumor area outlined by the TMD map (especially in the boundaries between tumor and the surrounding normal tissue) must be performed as well as to correlate the results with the MRI information in order to know if the tumor infiltration into normal brain tissue can be properly identified by the system. Additionally, through clinical validation, the relation between the improvement of the patient outcomes and the use of the system during the surgery will be studied.

## Figures and Tables

**Table 1:** Training dataset summary.

Class			#Patients	#Labelled Pixels
Normal			21	117,242
Tumor	Primary (G-IV)	GBM	8	12,641
	Primary (G-III)	Anaplastic Oligodendroglioma	2	1844
	Secondary	Lung	2	1936
		Renal	1	21
		Breast	1	325
Blood Vessel/Hypervascularized Tissue			22	57,429
Background			21	186,118
**Total (22 Patients, 36 Captures):**				**377,556**

**Table 2:** Validation HS image dataset characteristics.

Image ID	Size (MB)	#Pixels	Dimension (Width × Height × Bands)	Pathological Diagnosis
**P1C1**	362.62	224,770	495 × 456 × 826	Normal Brain
**P1C2**	197.90	122,670	471 × 262 × 826	Primary Grade II Oligodendroglioma
**P2C1**	225.35	139,682	332 × 423 × 826	Normal Brain
**P2C2**	276.99	171,699	364 × 474 × 826	Primary GBM
**P3C1**	402.26	249,344	513 × 488 × 826	Normal Brain
**P3C2**	230.34	143,560	485 × 296 × 826	Primary GBM
**P4C1**	372.47	230,878	480 × 483 × 826	Primary Grade I Meningioma

**Table 3:** Acquisition and processing time comparison between the sequential (Seq.) and accelerated (Acc.) implementations of the proposed algorithm for different size of images.

Image ID	Processing Type	Acquisition Time (s)	Pre-Processing (s)	Transmission (s)	PCA + SVM (s)	KNN (s)	HKM (s)	MV (s)	Total Processing Time (s)
P1C1	Seq.	19.98	15.07	0.00	11.32	378.87	39.68	0.009	444.95
	Acc.			14.00	6.02	8.16			68.76 *
	Speedup	N/A ^¥^	N/A ^¥^	0.00	1.88	46.45	N/A ^¥^	N/A ^¥^	6.47
P1C2	Seq.	19.02	6.50	0.00	5.90	196.64	21.87	0.004	230.92
	Acc.			7.15	4.35	4.23			35.53 *
	Speedup	N/A ^¥^	N/A ^¥^	0.00	1.36	46.44	N/A ^¥^	N/A ^¥^	6.50
P2C1	Seq.	13.40	9.35	0.00	6.72	158.66	24.96	0.005	199.70
	Acc.			8.07	4.48	3.48			42.38 *
	Speedup	N/A ^¥^	N/A ^¥^	0.00	1.50	45.62	N/A ^¥^	N/A ^¥^	4.71
P2C2	Seq.	14.70	12.59	0.00	8.96	212.96	30.45	0.006	264.97
	Acc.			9.56	5.02	4.66			52.61 *
	Speedup	N/A ^¥^	N/A ^¥^	0.00	1.78	45.74	N/A ^¥^	N/A ^¥^	5.04
P3C1	Seq.	20.71	19.72	0.00	13.68	434.96	44.57	0.008	512.93
	Acc.			13.34	6.72	9.44			77.63 *
	Speedup	N/A ^¥^	N/A ^¥^	0.00	2.03	46.10	N/A ^¥^	N/A ^¥^	6.61
P3C2	Seq.	19.58	8.94	0.00	7.73	234.90	25.75	0.005	277.33
	Acc.			9.45	4.66	5.08			44.15 *
	Speedup	N/A ^¥^	N/A ^¥^	0.00	1.66	46.27	N/A ^¥^	N/A ^¥^	6.28
P4C1	Seq.	19.38	13.84	0.00	11.49	377.60	41.59	0.007	444.52
	Acc.			12.36	6.29	8.15			67.79 *
	Speedup	N/A ^¥^	N/A ^¥^	0.00	1.83	46.34	N/A ^¥^	N/A ^¥^	6.56

* The total time obtained in the accelerated version is computed taking into account the maximum time obtained between the spatial-spectral supervised classification and the unsupervised clustering; ^¥^ Measurement not available.
